# Prevalence of Hangover Resistance According to Two Methods for Calculating Estimated Blood Alcohol Concentration (eBAC)

**DOI:** 10.3390/jcm9092823

**Published:** 2020-08-31

**Authors:** Chantal Terpstra, Andrew Scholey, Joris C. Verster, Sarah Benson

**Affiliations:** 1 Centre for Human Psychopharmacology, Swinburne University, Melbourne, VIC 3122, Australia; cterpstra@swin.edu.au (C.T.); andrew@scholeylab.com (A.S.); 2 Division of Pharmacology, Utrecht Institute for Pharmaceutical Sciences (UIPS), Utrecht University, 3584 CG Utrecht, The Netherlands; J.C.Verster@uu.nl

**Keywords:** hangover, alcohol, BAC, hangover resistance

## Abstract

Hangover resistance may be linked to an increased risk of continuing harmful drinking behaviours as well as involvement in potentially dangerous daily activities such as driving while hungover, mainly due to the absence of negative consequences (i.e., hangover symptoms) the day after alcohol consumption. The aim of this study was to examine the occurrence of claimed alcohol hangover resistance relative to estimated blood alcohol concentration (eBAC). A total of 1198 participants completed an online survey by answering questions regarding their demographics, alcohol consumption and occurrence of hangover. Two methods were used to calculate eBAC, one based on the modified Widmark Equation (N = 955) and the other from an equation averaging the total body water (TBW) estimates of Forrest, Watson, Seidl, Widmark and Ulrich (males only) (N = 942). The percentage of participants who claimed to be hangover resistant decreased rapidly with increasing eBAC and only a small number of hangover resistant drinkers remained at higher eBACs. Comparisons of the eBACs calculated by the two methods revealed significantly higher BACs when using the modified Widmark equation. These findings suggest that additional research for eBAC calculations is needed to improve accuracy and comprehensiveness of these equations for future alcohol hangover research.

## 1. Introduction

Alcohol hangovers are often the result of a night of heavy drinking and are a familiar phenomenon worldwide. The alcohol hangover refers to the combination of negative mental and physical symptoms, which can be experienced after a single episode of alcohol consumption, starting when blood alcohol concentration (BAC) approaches zero [1]. Historical definitions suggested that a blood alcohol concentration level equivalent to 0.11% was required to experience a next day hangover, with a greater percentage of drinkers reporting hangovers at higher BAC levels [2]. However, recent research shows that a considerable number of participants also reported hangover occurrence at moderately lower BAC levels [3]. The most reported hangover symptoms typically include fatigue [4,5], thirst [5], drowsiness [5], headache [5,6], dry mouth [5], nausea [7], reduced alertness [5], weakness [5], and concentration problems [5,8,9]. Other symptoms include vomiting, regret, heart racing, and apathy [5,10].

A hangover can result in impairment in both cognitive and psychomotor functioning [11]. This potential impairment can have repercussions when it comes to daily activities, such as driving a car, studying, or working [12]. A hangover can result in reduced productivity as individuals may be more likely to call in sick for work or study, or work at a reduced level of productivity. Employees with an alcohol hangover reported having significantly more conflicts with supervisors and co-workers, feeling miserable, or falling asleep on the job [13,14]. According to a large survey in The Netherlands, University students are less productive, and half of the students reported not being able to study while hungover. On average, students experience a hangover 2.7 days per month, which adds up to a total study loss of one month per year [15].

Because of the substantial impact of alcohol hangovers on health, economy, and society, it is important to determine the causes of alcohol hangover as well as factors that contribute to symptom severity. Recent research showed that hangover-resistant drinkers may be at increased risk of continuing harmful drinking behaviours as well as involvement in potentially dangerous daily activities, such as driving, mainly because of absence of negative consequences (i.e., severe hangover symptoms) the day after drinking [16,17]. Lower perceptions of hangover severity (thus experiencing less severe hangover symptoms) are associated with stronger beliefs that it is safe to drive the morning after drinking [18]. Despite the belief that one is safe to drive, research showed significant impairment in driving abilities while experiencing a hangover [19].

Identifying how hangover-resistant drinkers differ from non-hangover resistant drinkers may help to unfold the pathology of alcohol hangovers. Thus far, several theories exist as to why hangovers occur and what factors might influence severity, but this phenomenon remains largely unclear [20]. Previous research showed that the presence and severity of alcohol hangovers can differ between and within drinkers since the alcohol hangover is influenced by several other factors other than the amount of alcohol consumed. These factors include, but are not limited to, gender, age, personality, genetics, and health-related behaviours such as smoking, illicit drug use, sleep quality and duration as well as the type of drink consumed [21].

Quantity of alcohol consumed directly impacts the severity of hangovers and associated impairments [22]. As such, it is essential that studies assessing the effects of hangover collect and report data describing alcohol intake. Objective measures of alcohol concentration—such as those collected in blood, breath, saliva, and urine samples—can be obtained relatively easily in laboratory studies. However, when using a naturalistic design this is seldom accessible, as typically the researchers are not present during the drinking session. The naturalistic design in alcohol hangover research describes a methodology where participants consume alcohol on a typical night out and attend the laboratory for testing the following morning. The results are then compared to a non-hangover visit. Participants are questioned regarding their alcohol consumption from the night before, after which estimated blood alcohol concentration (eBAC) is calculated. While forgoing some of the experimental control inherent in laboratory testing, the naturalistic design allows an environment of less interference and free alcohol consumption compared to the typically smaller doses of alcohol approved for controlled studies [23]. Naturalistic studies are likely the most optimum to study hangovers, but it is also necessary to control several intervening factors that complicate the comparison of results among hangover studies. For example, in naturalistic designs, it is difficult to determine when BAC returns to 0. As alcohol hangover commonly utilizes naturalistic designs to ensure ecological validity, it is imperative that methodology to assess (naturalistic) hangovers adapts and develops according to existing information about hangovers [24]. Of relevance to the current study is the fact that the data needed to reconstruct BAC typically involves the number of alcoholic drinks consumed, duration of alcohol consumption, along with morphometric and demographic information such as gender, age, height, and weight [25].

The most widely used eBAC calculation was developed by Widmark [26,27]. This equation is dependent on the amount of alcohol consumed, the relative body water (set value for males (0.68) and females (0.55)), body mass (weight in kg), degradation rate in g/l (0.15), duration of alcohol consumption, and a set value for absorption time of 0.5 h. Please see Appendix A for additional information.

This equation has been modified as research has shown that the original Widmark formula had a tendency to overestimate BAC [25]. Specifically, the modified formula optimised the calculation of total amount of body water and included height and age [28]. The updated equations are found to be reasonably accurate for eBACs under controlled laboratory environments [28,29,30] and are frequently used in present alcohol hangover research to estimate BACs [3,31,32,33]. Please refer to Appendix B for additional information.

However, the use of eBAC has limitations when applied to real world drinking experiences. It is likely to introduce more variability of BAC and the accuracy of eBAC equations in naturalistic settings is relatively unknown [25]. Additionally, when reliant on self-report data from intoxicated participants, or retrospectively when remembering intoxication, accuracy of eBAC could be compromised due to poor recall [34]. Furthermore, eBAC calculations based on self-report data are less accurate when estimating higher BACs [35]. Therefore, it is important to compare eBAC calculations to examine whether different calculations are consistent or whether they may differentially affect results.

Approximately 20–25% of alcohol consumers are classed as ‘hangover resistant’, in that they report no hangover symptoms after a night of heavy drinking [36]. Previous research shows that hangover resistance is tightly coupled to eBAC levels [37,38]. Clearly, the number of hangover resistance claims is highly dependent on the amount of alcohol consumed; as the more drinks are consumed (and the higher the eBAC), the less likely drinkers are to claim hangover resistance. For example, with an eBAC level above 0.20%, only 8.1% of alcohol consumers claim hangover resistance [37].

Another factor that needs to be considered when investigating hangover resistance is the definition that is being used. A Canadian study used two definitions to describe hangover resistance. The first definition stated ‘Never having had a hangover during one’s lifetime’ and the second definition stated ‘Never having had a hangover during a certain time period (e.g., 1, 3, 5 years)’ [39]. A recent study showed that lifetime hangover resistance amongst heavier drinkers (eBAC 80 mg/dl, heaviest drinking occasion of the past month) occurs in 5–6% of participants [38]. Among people who claim hangover resistance, the symptoms reported by this group of heavy drinkers were limited to sleepiness and tiredness which were rated as less severe than the same items in the hangover sensitive group [40]. Even though hangover resistance seems to be rare and decreases with eBAC, a small number of people persist in claiming hangover resistance.

Due to the frequent use of eBAC in alcohol hangover research, it is vital to explore accuracy, comprehensiveness, and possible improvements for currently used formulas. This study aimed to assess whether the previously discussed calculations differ and explores the use of an additional and more comprehensive eBAC evaluation when assessing hangover resistance to explore similarities and differences in eBAC outcomes within an Australian population when compared with a previously used calculation in international research. This additional and more comprehensive method calculates eBAC by averaging the total body water (TBW) estimates of Forrest [41], Watson [28], Seidl [42], Widmark [26], and Ulrich [43] (males only) calculations. The mean TBW was then used in the following eBAC formula

BAC = (G/(TBW) − β × t(1)

where G is the amount of alcohol consumed in grams; β is the metabolic rate in grams per hour; and t is time in hours [44].

In line with previous research, it was predicted that around 50% of the participants would have relatively low eBACs (e.g., <0.08%) [37]. It was further predicted that higher eBACs will lead to a decrease of hangover resistance claims. Despite the decrease of hangover resistance prevalence with higher eBACs, we hypothesised that a small number of hangover resistance would persist. Additionally, the primary aim of the study was to evaluate, for the first time, the use of a more comprehensive eBAC methodology in the context of hangover resistance, and in particular to examine whether it produced different values to previous research.

## 2. Experimental Section

### 2.1. Method

The study was approved by the Swinburne University Human Research Ethics Committee (Reference 2012/045) and was conducted in accordance with the Declaration of Helsinki.

### 2.2. Design

This was an online survey assessing alcohol consumption behaviours and hangover resistance occurrence amongst an Australian population with the use of a commonly used method to calculate eBAC as well as an additional and more comprehensive calculation for eBAC.

### 2.3. Participants

Overall, 1748 respondents opened the online anonymous questionnaire. Data provided by non-drinkers were removed from the dataset. In total, 1198 participants completed the online survey (N = 444 male and N = 754 female), mostly consisting of students (65.3%). The mean age of the participants was 23.10 years old (SD = 4.68, range = 18–40 years old).

### 2.4. Measures

#### 2.4.1. Demographics

Participants answered questions regarding demographics (gender, age, weight, height), use of medications, tobacco, and illicit drugs.

#### 2.4.2. Alcohol Consumption Questions

Participants were questioned on their alcohol intake (e.g., in the past 30 days and the past 12 months). Alcohol consumption was defined using standardized Australian alcohol units (one standard drink = 10 g of pure alcohol). The consumption questions assessed frequency and quantity of alcohol consumed across various timescales (i.e., one occasion, 30 days, and 12 months) for each type of drink (beer, wine, spirits, and alcohol mixed with energy drink). Specifically, participants were asked the number of standard drinks and the number of hours that they had spent drinking.

#### 2.4.3. Estimated BAC (eBAC)

Responses to alcohol consumption questions regarding the heaviest drinking session within the previous 30 days were used to calculate eBAC with two different methods. Both methods computed the eBAC separately for males and females. Method 1 is the most frequently used method in previous research and is a modified Widmark equation [26]. The modified Widmark equation considers the number of alcoholic drinks, relative body water volume, weight, gender, and time needed to clear alcohol through metabolism [27], please see Appendix B for additional information [26]. Method 2 calculates eBAC by averaging the total body water (TBW) estimates of Forrest [41], Watson [28] Seidl [42], Widmark [26], and Ulrich [43] (males only) calculations. The mean TBW was then used in the following eBAC formula (1) in Section 1.

#### 2.4.4. Single Item Hangover Sensitivity/Resistance

Two groups were created, depending on the answer to the question: “I have had a hangover (headache, sick stomach) the morning after I had been drinking” over the past year. If the answer was ‘yes’, the participant was assigned to the hangover sensitive group. If the answer was ‘no’, the participant was assigned to the hangover resistant group.

### 2.5. Procedure

Participants were recruited via word of mouth, flyers, and advertisements on social media. Participation was voluntary and anonymous. Participants provided informed consent by agreeing to the survey terms online, after which they completed the online questionnaire. On completion of the survey, participants could choose to go into the draw to win one of two iPads by entering their email address.

### 2.6. Statistical Analysis

Data were collected online using SurveyMonkey and analysed using the Statistical Package for the Social Sciences version 25 (SPSS Inc., Chicago, IL, USA). Initially, the data were screened for any participants who did not meet the criteria, i.e., surveys completed between 2:00 a.m. and 7:00 a.m., and surveys submitted from the same IP address. Finally, any participant who answered ‘no’ to the final question, whether they had honestly and correctly answered all questions, were excluded. The mean, standard deviation and frequency distributions were calculated for the hangover sensitive group and the hangover resistant group. Comparisons between the two methods to calculate eBAC were made using nonparametric tests, since the eBAC data were skewed. Pearson’s Chi square test was used to measure associations between the different eBAC levels of the two methods and hangover resistance. Effects were regarded as statistically significant if *p* < 0.05.

## 3. Results

Two methods were used to calculate eBAC. First, the most commonly used method in previous alcohol hangover research was used and described, Method 1 [26,27]. Second, a more comprehensive method based on TBW was calculated (Method 2). Since results differed slightly depending on the eBAC method used, data are described separately.

Participants aged 36 years and older were excluded, since this question was assessed in a multiple-choice format and the exact age was needed in the eBAC calculation. This resulted in the removal of 100 participants, leaving 1098 participants. Participants with an estimated eBAC of 0.00% were excluded in both methods used because this suggests either no or extremely low alcohol consumption. Participants with an estimated eBAC of 0.40% and higher were also excluded in both methods, as this is an unreliably high estimation which suggests overestimation of the alcohol consumption and thus eBAC. This resulted in 20.3% of participant exclusion in Method 1 (leaving 955/1198) and 21.4% in Method 2 (leaving 942/1198). A general description of the typical drinking pattern is provided in Table 1.

### 3.1. Method 1

Of N = 955 remaining participants, 196 (20.5%) reported no hangover the day after drinking while 759 (79.5%) reported experiencing a hangover.

Consistent with previous research [37], participants were divided into seven groups, based on eBAC, to examine the occurrence of reported hangover resistance the day after drinking (Table 2). These eBAC values include those corresponding to the two most common internationally recognized legal driving limits (i.e., 0.05% and 0.08%) and the highest eBAC value (i.e., 0.20% and higher) as described in previous research [37].

The data show that 39.9 of drinkers fell within an eBAC below 0.08%. When considering only the subset of drinkers with eBACs above 0.08% the proportion of this subset who claimed hangover resistance was 11.5% (65/567). When the smaller subset of drinkers with eBACs above 0.20% was considered, the prevalence of hangover resistance was 12.5% (21/168), respectively.

### 3.2. Method 2

**Figure 1:**
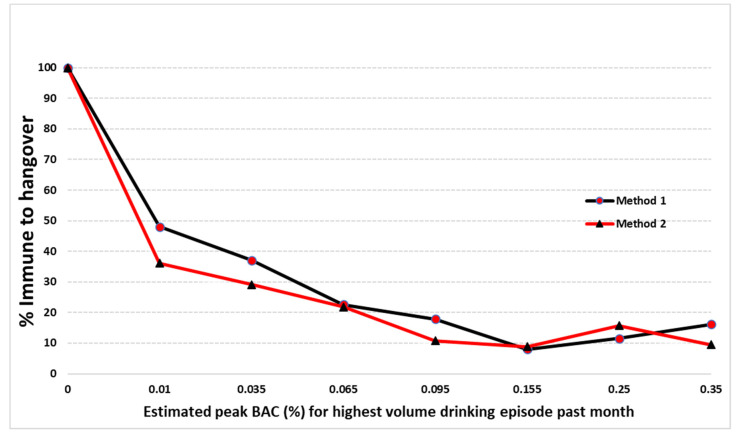
Prevalence of hangover resistance after alcohol consumption at different eBAC levels for Method 1 [26] and Method 2 [44]. *p* < 0.05 with Chi Square test of independence.

Of N = 942 remaining participants, 19.9% reported no hangover symptoms the day after drinking (N = 187) and 755 (80.1%) reported experiencing hangover symptoms. The participants were divided into seven groups to examine the occurrence of hangover resistance the day after drinking within different eBAC ranges, which are shown in Table 2. This is consistent with previous research [37] to be able to compare our findings. These eBAC values correspond to the two most common internationally recognized legal driving limits (i.e., 0.05% and 0.08%) and the highest eBAC value (i.e., 0.20% and higher) [37]. The frequency distributions for hangover resistant drinkers across the eBAC continuum of Method 2 are also shown in Figure 1.

The data shows that 49.4% of drinkers fell within an eBAC below 0.08%. When considering only the subset of drinkers with eBACs above 0.08% the proportion of this subset who claimed no next day adverse effects after drinking was 10.7% (51/476). When the smaller subset of drinkers with eBACs above 0.20% was considered, the prevalence of no adverse effects the day after drinking was 14.5% (16/110), respectively.

### 3.3. Comparison of Two Methods

eBAC data was skewed for both eBAC methods, since most people are distributed along the lower eBAC ranges. Therefore, a Wilcoxon signed-rank test, which evaluated the difference between medians for eBAC Method 1 and eBAC Method 2 was conducted. The results indicated that eBAC levels of Method 1 were statistically significantly higher than eBAC levels of Method 2, *z* = −11.07, *p* < 0.001.

A chi-square test of independence showed that there were no significant differences in reported hangover resistance in the two eBAC methods and across the 7 BAC groups [45]. Results of the chi-square test can be found in Table 2.

## 4. Discussion

The current study assessed the occurrence of hangover resistance amongst an Australian population and compared two eBAC equations. Several of our hypotheses were supported. Both eBAC methods showed that almost half of the drinkers were distributed at eBAC levels below 0.08% (Method 1: 39.9% and Method 2: 49.4%), which supports the findings of previous research [37,38]. Previous research found that only a small percentage of 8.1% of participants who did not have a hangover in the past year were distributed at an eBAC level of 0.20% and higher [37]. A Canadian study found an even lower level of 4–5% of hangover resistance at an eBAC level of 0.20% and higher [38].

The present study confirms that the percentage of people who claim to be hangover resistant decreases with increasing eBACs, independent of eBAC method. However, contrary to previous research, a slight increase of hangover resistance claims occurred above 0.20% in the present study for both eBAC methods. This is likely a chance fluctuation due to smaller numbers of participants claiming hangover resistance at higher eBACs [36,37,40].

When comparing the two methods to calculate eBAC, several similarities were found. Both methods were able to show a decrease in hangover resistance prevalence with higher eBACs. Both methods showed that between 22–25% of the drinkers reported no hangover, however almost half of these drinkers were distributed at eBAC levels below 0.08%, which is a threshold to determine impaired driving in several countries [37].

The differences between the two methods are found in the mean eBACs, with a significantly higher mean eBAC for Method 1 than for Method 2. Interestingly, hangover resistance prevalence in several eBAC increments was observed to be slightly, but not significantly, lower with the use of Method 2. An explanation for this could possibly be found in the notion that previous research found that Method 1 (based on a modified Widmark formula) [26,27] might lead to an overestimation of eBAC when used in naturalistic settings with the help of self-report measures [29].

Differences found between the two eBAC methods used in this study suggest that additional research exploring the accuracy and comprehensiveness of eBAC equations is needed for future (naturalistic) hangover research. As illustrated earlier, previous alcohol hangover research most commonly uses the modified Widmark calculations for BAC [3,31,32,33]. Previous research suggests that higher levels of BAC (0.11%) were needed to develop a next day hangover. However, a recent study suggests that hangover prevalence and severity occur at lower BACs [3]. This may mean that previous alcohol hangover research overestimated BACs and that hangover occurrence and severity occur at lower BACs. Interestingly, a significant difference was found between the hangover resistant and hangover sensitive group on all questions related to alcohol consumption with numerically higher means in the hangover sensitive group. This suggests that even though hangover occurs at lower BACs [3], BAC is a relevant indicator for hangover occurrence. Based on this study only, there appear to be no clinically relevant differences between the two methods of eBAC calculation.

Limitations of this study include relying on self-reported alcohol intake and eBAC calculations to estimate BAC obtained during the drinking episode. Heavy drinking often results in memory impairment, which may lead to less accuracy and recall-bias [46]. This limitation is often observed in (semi) naturalistic hangover research. However, it can be argued that the naturalistic design of this study relates to a representative alcohol consumption in real life situations.

Objective measures of ethanol assessments are absent in this study, which could pose the question whether the calculations for both BAC methods are accurate. It is unclear whether similar results would be found in controlled settings based on these study findings. However, the main purpose of this study was to assess whether the two methods to calculate eBAC differ rather than assess the accuracy of the two methods. An objective measure of ethanol assessment, such as blood and saliva, should be utilized for future research purposes. A controlled setting would allow equal quantity of alcohol consumption in both groups of drinkers. This ensures valid comparison of hangover-resistant and non-hangover-resistant drinkers in a controlled setting, which could then be translated to naturalistic designs. Including measures on hangover frequency and severity in future research will enhance our understanding of differences between hangover-resistant and non-hangover resistant drinkers.

## 5. Conclusions

The findings presented here show that almost half of drinkers had an eBAC below 0.08%, irrespective of applying Method 1 or Method 2 to calculate estimated BAC. The present results support previous findings and show that the percentage of people who claim to be hangover resistant decreases rapidly with increasing eBACs. However, a small number of hangover resistant drinkers persists, independently of higher eBACs. Differences between the two eBAC methods are found in the mean eBACs, with a significantly higher mean eBAC for Method 1. Future research should assess influences of eBAC on hangover resistance prevalence with the use of an objective and more extensive assessment of hangover (severity) and should further explore research on eBAC calculations to improve their accuracy and comprehensiveness for future research. Notwithstanding the absence of significant findings, this study highlights the importance of continuing assessment of the methodology used to assess hangovers to increase knowledge of alcohol hangover resistance and pathology.

## Figures and Tables

**Table 1:** Participant morphometrics, demographics, and drinking characteristics. Numbers are means with standard deviations in parentheses. * *p* < 0.05 between hangover resistant and hangover sensitive group of participants.

	Full Sample	Hangover Resistant	Hangover Sensitive
Age	23.10(4.7)	22.95(5.1)	23.14(4.6)
Height (m)	1.71(0.1)	1.69(0.1) *	1.71(0.1) *
Weight (kg)	70.57(15.6)	69.93(17.7)	70.77(15.0)
Standard drinks per occasion	5.25(3.9)	3.40(2.6) *	5.83(4.1) *
Days used alcohol (30 days)	6.97(6.7)	4.93(6.1) *	7.60(6.8) *
Days drunk (30 days)	2.43(3.5)	0.95(2.4) *	2.90(3.6) *
Days binge (30 days)	3.22(4.3)	1.50(3.5) *	3.76(4.4) *
Greatest number of drinks (30 days)	7.87(6.1)	4.72(4.8) *	8.85(6.1) *
Consumption duration (hours)	5.16(3.5)	3.68(3.0) *	5.62(3.5) *
Alcohol consumed (grams)	78.67(61.1)	47.23(48.1) *	88.54(61.4) *
eBAC (Method 1) (N = 955) eBAC (Method 2) (N= 942)	0.12(0.1) 0.10(0.1)	0.08(0.1) * 0.07(0.1) *	0.13(0.1) * 0.11(0.1) *

**Table 2:** Percentage of participants who report no hangover symptoms following alcohol consumption across various eBAC ranges for Method 1 and Method 2.

	eBAC Range	% No Hangover Method 1	% No Hangover Method 2	*χ* ^2^	*p*
Group 1	0 ≤ BAC < 0.02%	48.0 (36/75)	36.1 (53/147)	2.95	0.09
Group 2	0.02% ≤ BAC < 0.05%	37.1 (62/167)	29.1 (53/182)	2.53	0.11
Group 3	0.05% ≤ BAC < 0.08%	22.6 (33/146)	21.9 (30/137)	0.02	0.89
Group 4	0.08% ≤ BAC < 0.11%	17.9 (22/123)	10.8 (13/120)	2.45	0.12
Group 5	0.11 ≤ BAC < 0.20%	8.0 (22/275)	8.9 (22/246)	0.15	0.70
Group 6	0.20 ≤ BAC < 0.30%	11.5 (15/131)	15.7 (14/89)	0.85	0.36
Group 7	0.30 ≤ BAC < 0.40%	16.2 (6/37)	9.5 (2/21)	0.50	0.48

Note. This table also shows results of a chi-square test of independence to investigate associations between the two methods and hangover resistance.

## Appendix A. Widmark Formula

BAC = A/(r × G) − B × (t − 0.5),

BAC = blood alcohol concentration in g/L, A = number of standard drinks consumed in grams, r = relative amount of body water (l/kg), G = weight of subject in kg, B = degradation rate in g/L of 0.15, t = elapsed time during alcohol consumption in hours, r = a set value for males (0.68) and females (0.55), the absorption time is 0.5 h.

## Appendix B. eBAC Calculation Method 1

$$C=\frac{A}{s+(u\times G)}-B\times (t-0.5)$$

*C* = blood alcohol concentration in g/L, *A* = alcohol consumed in grams, *s* = set value for males (17.45) and females (18.075), *u* = set value for males (0.4786) and females (0.3186), *G* = weight of subject in kg, *B* = degradation rate in g/L of 0.15, *t* = elapsed time during alcohol consumption in hours, the absorption time is 0.5 h.
