# Correction: Terpstra et al. Prevalence of Hangover Resistance According to Two Methods for Calculating Estimated Blood Alcohol Concentration (eBAC). *J. Clin. Med.* 2020, *9*, 2823

**DOI:** 10.3390/jcm11020293

**Published:** 2022-01-06

**Authors:** Chantal Terpstra, Andrew Scholey, Joris C. Verster, Sarah Benson

**Affiliations:** 1 Centre for Human Psychopharmacology, Swinburne University, Melbourne, VIC 3122, Australia; cterpstra@swin.edu.au (C.T.); andrew@scholeylab.com (A.S.); J.C.Verster@uu.nl (J.C.V.); 2 Division of Pharmacology, Utrecht Institute for Pharmaceutical Sciences (UIPS), Utrecht University, 3584 CG Utrecht, The Netherlands

The authors wish to make the following corrections to the original article [1]:

Appendix A, should be titled ***Widmark Formula*** and the following text removed:

For males rw = Total Body Water = 2.447 − 0.09515(y) + 0.1074(h) + 0.3362(W)

For females rw = Total Body Water = −2.097 + 0.1069(h) + 0.04666(W)

Appendix B, should be titled ***eBAC Calculation Method 1***. Also, the content should be replaced into:

$$C=\frac{A}{s+\left(u\times G\right)}\;-B\times \left(t-0.5\right)$$

*C* = blood alcohol concentration in g/L, *A* = alcohol consumed in grams, *s* = set value for males (17.45) and females (18.075), *u* = set value for males (0.4786) and females (0.3186), *G* = weight of subject in kg, *B* = degradation rate in g/L of 0.15, *t* = elapsed time during alcohol consumption in hours, the absorption time is 0.5 h.

The authors apologize to the readers for any inconvenience caused by these changes. It is important to note that these corrections do not affect the study results or interpretation. The original manuscript will remain online on the article webpage, with reference to this Correction.
